# Gemcitabine in Bone Sarcoma Resistant to Doxorubicin-Based Chemotherapy

**DOI:** 10.1155/S1357714X00000025

**Published:** 2000-03

**Authors:** Ofer Merimsky, Isaac Meller, Yehuda Kollender, Josephine Issakov, Gideon Flusser, Moshe Inbar

**Affiliations:** 1 Department of Oncology The Tel-Aviv Sourasky Medical Center Affiliated with Sackler Faculty of Medicine Tel-Aviv University Tel-Aviv Israel; 2 Department of The National Unit of Orthopedic Oncology The Tel-Aviv Sourasky Medical Center Affiliated with Sackler Faculty of Medicine Tel-Aviv University Tel-Aviv Israel; 3 Departments of Pathology The Tel-Aviv Sourasky Medical Center Affiliated with Sackler Faculty of Medicine Tel-Aviv University Tel-Aviv Israel; 4 Departments of Radiology The Tel-Aviv Sourasky Medical Center Affiliated with Sackler Faculty of Medicine Tel-Aviv University Tel-Aviv Israel

## Abstract

*Subjects and Methods:* Seven patients with progressive localized or metastatic chemo-resistant osteosarcoma were treated by gemcitabine.The protocol included gemcitabine 1000 mg/m2/w for 7 consecutive weeks, followed by 1 week rest. If no progression was observed,maintenance by gemcitabine 1000 mg/m2/w for 3 weeks every 28 days was given until failure was clinically or radiologically evident.

*Results.* The true objective response rate was 0%. However, disease stabilization and clinical benefit response were observed in five patients (70%) for 13–96 weeks.

*Discussion.* Postponing the inevitable death with a relatively non-toxic treatment, is, in our opinion, an important issue especially in young patients.Thus it may be justified and warranted to investigate the activity of gemcitabine in a larger group of patients with bone sarcomas.


*Sarcoma* (2000) **4**, 7–10

ORIGINAL ARTICLE

### Gemcitabine in bone sarcoma resistant to doxorubicin-based chemotherapy

OFER MERIMSKY,[1] ISAAC MELLER,[2] YEHUDA KOLLENDER,[2] JOSEPHINE ISSAKOV,[3] GIDEON FLUSSER[4] & MOSHE INBAR[1]

*Department of [1]Oncology, the [2]National Unit of Orthopedic Oncology, the Departments of [4]Radiology and [3]Pathology, The Tel-Aviv Sourasky Medical Center, Affiliated with Sackler Faculty of Medicine, Tel-Aviv University, Tel-Aviv, Israel*

**Abstract**

*Subjects and Methods*: Seven patients with progressive localized or metastatic chemo-resistant osteosarcoma were treated by gemcitabine. The protocol included gemcitabine 1000 mg/m2/w for 7 consecutive weeks, followed by 1 week rest. If no progression was observed, maintenance by gemcitabine 1000 mg/m2/w for 3 weeks every 28 days was given until failure was clinically or radiologically evident.

*Results.* The true objective response rate was 0%. However, disease stabilization and clinical benefit response were observed in five patients (70%) for 13–96 weeks.

*Discussion.* Postponing the inevitable death with a relatively non-toxic treatment, is, in our opinion, an important issue especially in young patients. Thus it may be justified and warranted to investigate the activity of gemcitabine in a larger group of patients with bone sarcomas.

**Key words**: *bone sarcoma, gemcitabine*

### Introduction

Gemcitabine hydrochloride is a pyrimidine nucleoside analog which is applied as a chemotherapeutic antimetabolite. Gemcitabine inhibits DNA replication by inhibiting DNA synthesis and by blocking repair mechanisms through masked chain termination. Additionally, gemcitabine exerts several other actions that self-potentiate its cytotoxic activity. Gemcitabine is usually well tolerated by the patients and its common associated side-effects are not severe, and include low grade myelotoxicity, flu-like syndrome, fever, rash, swelling of the legs, nausea and vomiting. Gemcitabine has demonstrated significant clinical activity and clinical benefit response in a variety of tumors.[1–4]

Very limited data is available on the use of gemcitabine in soft tissue (STS) or bone sarcomas. Gemcitabine was found to be active on xenograft of STS growing in nude mice.[5,6] Palliative effects of gemcitabine in a patient with osteosarcoma resistant to standard chemotherapy[7] and experience with gemcitabine in patients with a variety of progressive sarcomas[8] have been recently reported by our team. This report focuses on the effect of gemcitabine on bone sarcoma.

### Treatment protocol

Eligibility criteria were recurrent or metastatic osteosarcoma, that either failed to respond to standard chemotherapy for metastatic or recurrent disease and progressed while on chemotherapy, or relapsed after having been treated with pre-operative or adjuvant chemotherapy and had demonstrated progressive disease over a period of 3 months. Standard chemotherapy for bone sarcoma had to include methotrexate (MTX), cisplatin (CDDP), adriamycin ADR and ifosmamide IFX. All the patients had to be 15 years or older, with a Karnofsky's performance status (KPS) of at least 40%, and life expectancy of at least 3 months. Any bone sarcoma type was permitted provided that there was measurable disease, and there were no central nervous system nor spinal cord involvement. Signed informed consent was mandatory (in case of patients younger than 18, parents also signed).

Baseline evaluation included interview and assessment of symptoms severity and quality of life, measurement and documentation of marker lesions by CT scan, ultrasound or plain X-rays, and complete blood count and biochemical serum analysis.

Treatment consisted of induction by gemcitabine

Correspondence to: O. Merimsky, MD, Department of Oncology, The Tel-Aviv Sourasky Medical Center, 6 Weizman Street, Tel-Aviv 64239, Israel. Fax: Int-972-3-697-4828; E-mail: merimsky@zahav.net.il

1357-714X print/1369-1643 online/00/010007-04 © 2000 Taylor & Francis Ltd

1000 mg/m2/w for 7 consecutive weeks, followed by 1 week rest. Response to induction course was assessed by interview (for clinical benefit response and quality of life) and by repeated ancillary tests. If no progression was observed, maintenance by gemcitabine 1000 mg/m2/w for 3 weeks every 28 days was given until failure was clinically or radiologically evident. Evaluation of response, toxicity and quality of life, was performed every 3 months by interview, physical examination and ancillary tests, according to the WHO criteria. Progression was determined as deterioration in clinical symptoms, appearance of new lesions or enlargement of a lesion by at least 25% of its pre-treatment size. Treatment was to be stopped in case of life-threatening toxicity, progression of the disease, or on patient's refusal to continue.

### Patients

Seven patients with primary extremity bone sarcoma, at age range of 15–43 years, were enrolled from December 1996 through August 1999 into an ongoing phase II study on gemcitabine in soft tissue or bone sarcoma.[8] All the patients were heavily pre-treated by various agents according to their disease, such as adriamycin, ifosfamide, high-dose ifosfamide, methotrexate and etoposide. The involved sites were mainly the local tumor bed and lung. The main symptoms were pain and respiratory problems. Patient characteristics are detailed in Table 1.

### Results

The true objective response rate of osteosarcoma to gemcitabine was 0%. However, disease stabilization was observed in 5 out of 7 patients after having failed on previous treatments. Time to progression varied from 13 to 96 weeks. It should be noted that disease stabilization was accompanied by clinical benefit response, i.e. improvement of performance status, alleviation of respiratory symptoms, alleviation of pain and reduction in narcotics consumption) and was observed only in those who also achieved a progression-free state.

All the patients who failed to respond to gemcitabine did not have any clinical benefit response. The treatment was well tolerated by the patients. Hematological toxicity was the main concern in our patients, of whom the vast majority was heavily pre-treated. Other toxic effects included weakness, rash ascites (with no malignant cells in repeated taps), limb edema (deep vein thrombosis was excluded by Doppler-ultrasound study), and low grade fever.

### Discussion

Bone sarcomas carry poor prognosis. Close to one half of patients succumb to metastatic or locally advanced disease. Metastatic sarcoma is usually fatal and treatment options are rather limited. Median survival from the time metastases are detected is relatively short, although 20–25% of patients with metastatic sarcoma are alive 2 years after diagnosis.

Patients with metastatic sarcoma often are asymptomatic at the time that a radiograph or CT reveals metastases, and may remain free of symptoms for long periods of time. Thus, alleviation of symptoms is not an immediate concern in many patients, although disease progression is eventually inevitable.

Numerous drug combinations have been assessed in treated and untreated metastatic disease. The most effective of these have contained cisplatin, doxorubicin and high-dose methotrexate–leucovorin factor either as a two- or three-drug regimen; response rates of the order of 25–35% have been obtained, although often based on rather small numbers. The most important studies on palliative chemotherapy in metastatic osteosarcoma include cyclophosphamide+ doxorubicin+dacarbazine (29 patients, response rate 24%[9]), cisplatin + vincristine+high-dose methotrexate (29 patients, response rate 28%[10]), dacarbazine+doxorubicin D (20 patients, response rate 35%[11], Dacarbazine+doxorubicin (19 patients, response rate 26%[12]), and cyclophosphamide+ doxorubicin+actinomycin D (20 patients, response rate 25%[9]). The oncological problem starts after failure of these agents to affect the disease course. The patients, especially the younger, may still have considerable life expectancy and good performance status, and are eager to be treated, but the caring oncologist may have nothing to propose. There is a need for active and minimally toxic agents that can be given as second line treatment in patients with STS or bone sarcoma. This was the rationale to use gemcitabine in this hopeless population of patients with sarcoma.

The information given to patients before participation in such study, especially when no more standard therapy exists for young patient with strong life will, should give 'controlled hope' but without illusions for cure. The patients were told that gemcitabine was highly experimental in osteosarcoma, that there was no literature on the topic, that the worldwide experience with the drug was achieved in other diseases, and that the expected toxicity was relatively mild.

Our results pointed to the important effect of gemcitabine treatment in heavily pre-treated patients with progressive bone sarcomas. Gemcitabine was found to be effective in achieving stabilization of osteosarcoma refractory to standard chemotherapy consisting mainly of adriamycin, high-dose methotrexate, cisplatin and ifosfamide. Although disease stabilization is generally accepted as failure of chemotherapy, in this series of cases it should be regarded as success due to noteworthy disease control, clinical benefit response and low toxicity profile, especially in view of failure of the accepted drugs.

It is interesting to note that gemcitabine has shown activity in STS. In a recent study reported by our

**Table 1**. *Patient characteristics*

| Sex | Age | Site | Histology | Previous treatments | Disease progression in: | KPS | Symptoms | Response to gemcitabine | TTP (w) |
|---|---|---|---|---|---|---|---|---|---|
| m | 24 | Femur | os | CDDP, ADR, MTX, HD-IFX | Lung, local recurrence | 40 | Pain, dyspnea, weakness | lung: PD local recurrence: PD | 0 |
| m | 25 | Femur | os | CDDP, ADR, MTX, HD-IFX, ICE | Lung | 70 | Pain, cough, weakness | lung: PD local recurrence: PD | 0 |
| m | 18 | Femur | os | CDDP, ADR, MTX, VP16, IFX, Bleo, CTX, ACT-D, surgery | Lung, bone | 70 | Pain, weak, nausea, fatigue | CBR lung, bone: SD | 17 |
| m | 22 | Femur | os | CDDP, ADR, MTX, HD-IFX, surgery | Lung | 80 | Cough, pain | CBR lung SD | 42 |
| f | 26 | Pubis | os | CDDP, ADR, MTX, HD-IFX, VP16, surgery | Local recurrence | 50 | Pain, Limb edema, fever | CBR local recurrence: SD | 96 |
| f | 15 | Femur | os anaplastic | CDDP, ADR, MTX, IFX, VP16 | Local recurrence | 50 | Pain, limb edema; | CBR: less pain, KPS 80, SD | 13 |
| m | 43 | Tibia | os leiomyosarcoma | CDDP, ADR, MAID, surgery, RT, HD-IFX, VP16 | Bones | 70 | Pain, weakness | CBR: less pain, bone SD | 37 |

ACT-D, Actinomycin-D; ADR, adriamycin; Bleo, bleomycin; CDDP, cisplatin; CTX, cyclophosphamide; HDIFX, high dose ifosfamide (12 gm/$m^2$ given continuously over 72 h); ICE, ifosfamide, carboplatin, etoposide; IFX, ifostamide; MAID, mesna, adriamycin, ifosfamide, dacarbazine; MTX, methotrexate; OS, osteosarcoma; RT, radiation therapy; TTP (w), time to progression in weeks; VAC-VPI, protocol for Ewing's sarcoma: vincristine, adriamycin, cyclophosphamide alternating with etoposide and ifosfamide;
VP16, etoposide.

group[8] we have documented one partial response (leiomyosarcoma) and one minimal response (angiosarcoma), yielding an true objective response rate of 5.5%. An additional six patients achieved stabilization of disease, yielding an overall progression-free rate of 44%. The median time to progression was more than 27 weeks.

Patel *et al.*[13] reported their experience in patients with various types of STS that were given gemcitabine in a schedule similar to that in our trial. The best responses were observed, as in our study, in patients with angiosarcoma and leiomyosarcoma. The observations in these trials point to a possible role of gemcitabine in the treatment in angiosarcoma and leiomyosarcoma. In our study and in Patel's study, gemcitabine monotherapy was given in a schedule of 1 gr/m2/week for 3 weeks every 28 days. A different schedule is suggested by Spith-Schwalbe *et al.*:[14] 200 mg/$m^2$ given on days 1,8,15 by 6-h continuous infusion every 28 days. In their study, 11 heavily pre-treated patients with STS were enrolled. The side-effects were mainly hematological, and the response, i.e. two partial responses and three cases with disease stabilizations, were all in pulmonary metastases. Postponing the inevitable death with a relatively non-toxic treatment is an important issue especially in cases of young patients.

It is clear that no treatment recommendations can be made on the basis of such small series. However, it may be justified and warranted to investigate the activity of gemcitabine in a larger group of patients with bone sarcomas, even as a first line treatment for recurrent or metastatic disease.

### References

1 Carmichael J, Fink U, Russell RC, *et al.* Phase II study of gemcitabine in patients with advanced pancreatic cancer. *Br J Cancer* 1996;73:101–5.
2 Rothenberg ML, Moore MJ, Cripps MC, *et al.* A phase II trial of gemcitabine in patients with 5-FU refractory pancreas cancer. *Ann Oncol* 1996;7:347–53.
3 Abratt RP, Bezwoda WR, Falkson G, Goedhals L, Hacking D, Rugg TA. Efficacy and safety profile of gemcitabine in non-small-cell lung cancer: a phase II study. *J Clin Oncol* 1994;12:1535–40.
4 FDA. Quality of life matters!. News section. *Ann Oncol* 1995;6:854–60.
5 Braakhuis BJ, Ruiz van Haperen VW, Boven E, Veerman G, Peters GJ. Schedule dependent antitumor effect of gemcitabine *in vivo* model system. *Semin Oncol* 1995;22:42–46.
6 Boven E, Schipper H, Erkelens CA, Hatty SA, Pinedo HM. The influence of the schedule and the dose of gemcitabine on the anti-tumour efficacy in experimental human cancer. *Br J Cancer* 1993;68:52–6.
7 Merimsky O, Meller I, Kollender Y, Inbar M. Palliative effect of gemcitabine in osteosarcoma resistant to standard chemotherapy. *Eur J Cancer* 1998;34:1296–7.
8 Merimsky O, Meller I, Flusser G, *et al.* Gemcitabine in soft tissue or bone sarcoma resistant to standard chemotherapy: a phase II study. *Cancer Chemother Pharmacol* 2000;45:177–81.
9 Benjamin RS, Baker LH, O'Bryan RM, Moon TE, Gottlieb JA. Chemotherapy of metastatic osteosarcoma–studies by the M.D. Anderson Hospital and the Southwest Oncology Group. *Cancer Treatment Rep* 1978;62:237–8.
10 Morgan E, Baum E, Bleyer WA, *et al.* Treatment of patients with metastatic osteogenic sarcomas: a report from the Children's Cancer Study Group. *Cancer Treatment Rep* 1984;68:661–4.
11 Rosen G, *et al.* Cisplatin–adriamycin combination chemotherapy in evaluable osteogenic sarcoma. *Proc Am Soc Clin Oncol* 1982;1:173.
12 Pratt CB, Champion JE, Senzer N, *et al.* Treatment of unresectable or metastatic osteosarcoma with cisplatin or cisplatin–doxorubicin. *Cancer* 1985;56:1930–3.
13 Patel SR, Jenkins J, Papadopoulos NE, *et al.* Preliminary results of a two-arm phase 2 trial of gemcitabine (Gem) in patients (Pts) with gastrointestinal leiomyosarcoma and other soft tissue sarcomas (STS). *Proc Am Soc Clin Oncol* 1999;18:541a.
14 Spith-Schwalbe E, Koschuth A, Grunewald R, Genvresse I, Possinger K. Gemcitabine in pretreated patients with advanced STS: preliminary results from a phase II trial. *Proc Am Soc Clin Oncol* 1998;17:A1976.
